# How to (or Not to) Integrate Vertical Programmes for the Control of Major Neglected Tropical Diseases in Sub-Saharan Africa

**DOI:** 10.1371/journal.pntd.0000755

**Published:** 2010-06-29

**Authors:** Narcis B. Kabatereine, Mwele Malecela, Mounir Lado, Sam Zaramba, Olga Amiel, Jan H. Kolaczinski

**Affiliations:** 1 Vector Control Division, Ministry of Health, Government of Uganda, Kampala, Uganda; 2 Tanzania Lymphatic Filariasis Elimination Programme, National Institute for Medical Research, Dar es Salaam, Tanzania; 3 Directorate of Preventive Medicine, Ministry of Health, Government of Southern Sudan, Juba, Southern Sudan; 4 Director General, Health Services, Ministry of Health, Kampala, Uganda; 5 Ministry of Health, Government of Mozambique, Maputo, Mozambique; 6 Malaria Consortium—Africa Regional Office, Kampala, Uganda; 7 Disease Control and Vector Biology Unit, London School of Hygiene & Tropical Medicine, London, United Kingdom; London School of Hygiene & Tropical Medicine, United Kingdom

## Abstract

Combining the delivery of multiple health interventions has the potential to minimize costs and expand intervention coverage. Integration of mass drug administration is therefore being encouraged for delivery of preventive chemotherapy (PCT) to control onchocerciasis, lymphatic filariasis, schistosomiasis, soil-transmitted helminthiasis, and trachoma in sub-Saharan Africa, as there is considerable geographical overlap of these neglected tropical diseases (NTDs). With only a handful of countries having embarked on integrated NTD control, experience on how to develop and implement an efficient integrated programme is limited. Historically, national and global programmes were focused on the control of only one disease, usually through a comprehensive approach that involved several interventions including PCT. Overcoming the resulting disease-specific structures and thinking, and ensuring that the integrated programme is embedded within the existing health structures, pose considerable challenges to policy makers and implementers wishing to embark on integrated NTD control. By sharing experiences from Uganda, Tanzania, Southern Sudan, and Mozambique, this symposium article aims to outlines key challenges and solutions to assist countries in establishing efficient integrated NTD programmes.

## The Challenge

Resources for disease control are limited and thus need to be used efficiently [1]. This need is particularly apparent for the control of neglected tropical diseases (NTDs), which, until recently, was largely unfunded [2]. Integration of disease-specific programmes is therefore being encouraged for onchocerciasis, lymphatic filariasis (LF), schistosomiasis, soil-transmitted helminth (STH) infection, and trachoma [3], [4]. These NTDs occur over more or less the same areas and their control depends, although not exclusively, on regular mass drug administration (MDA) of safe and effective preventive chemotherapy (PCT) [5]; combined PCT delivery should thus minimize costs and increase coverage [6], [7]. However, “real world” experience of implementing such integrated NTD control is limited [8], [9], making it difficult to decide how best to embark on, and proceed with, the development of an integrated NTD control programme in a manner that promotes efficiency and local ownership—both prerequisites for sustainability.

## Tutorial

### What is integrated NTD control?

Integrated delivery of health services covers a range of approaches and the definition is therefore context dependent [10]–[12]. Here we focus on a group of NTDs for which regular MDA of PCT is key to effective control, namely onchocerciasis, LF, schistosomiasis, STH infection, and trachoma. In this context, integration is usually applied to creation of “PCT packages” by combining MDA for more than one NTD. Some countries have formed umbrella NTD programmes to oversee vertical delivery of these packages through campaigns or other channels. In other settings, a more “horizontal” approach is applied whereby intervention packages are delivered as part of routine health care and education programmes. Both approaches provide opportunities for “integration” [13], [14] and are by no means mutually exclusive [15]. Instead they should be coordinated and combined with the goal of maximising efficiency [16].

### What challenges should be anticipated?

Integrating components of control strategies for different diseases is technically and managerially challenging. It is good to appreciate these challenges at the outset, so as to be able to manage expectation of donors, programme managers, and beneficiaries, as well as the roles and responsibilities of a multitude of stakeholders. Clear leadership of an integrated programme will need to be established. In countries with existing disease-specific control programmes, this may mean combining similar roles and responsibilities of various programme managers under one position, which could result in redundancies. Alternatively, the programme leadership may be assigned to one individual while the other managers of formerly disease-specific programmes contribute to design and implementation of the integrated approach. In any case, such restructuring may cause resentment among managers, in extreme cases leading to obstruction and other difficulties in managing the process. Another human resource implication to be aware of is the need for substantial training. Staff at all levels will need to acquire new knowledge about the additional diseases they are now meant to control, and about the collection and interpretation of data to monitor and evaluate their integrated programmes.

### What preparatory activities are required?

**Figure 1:**
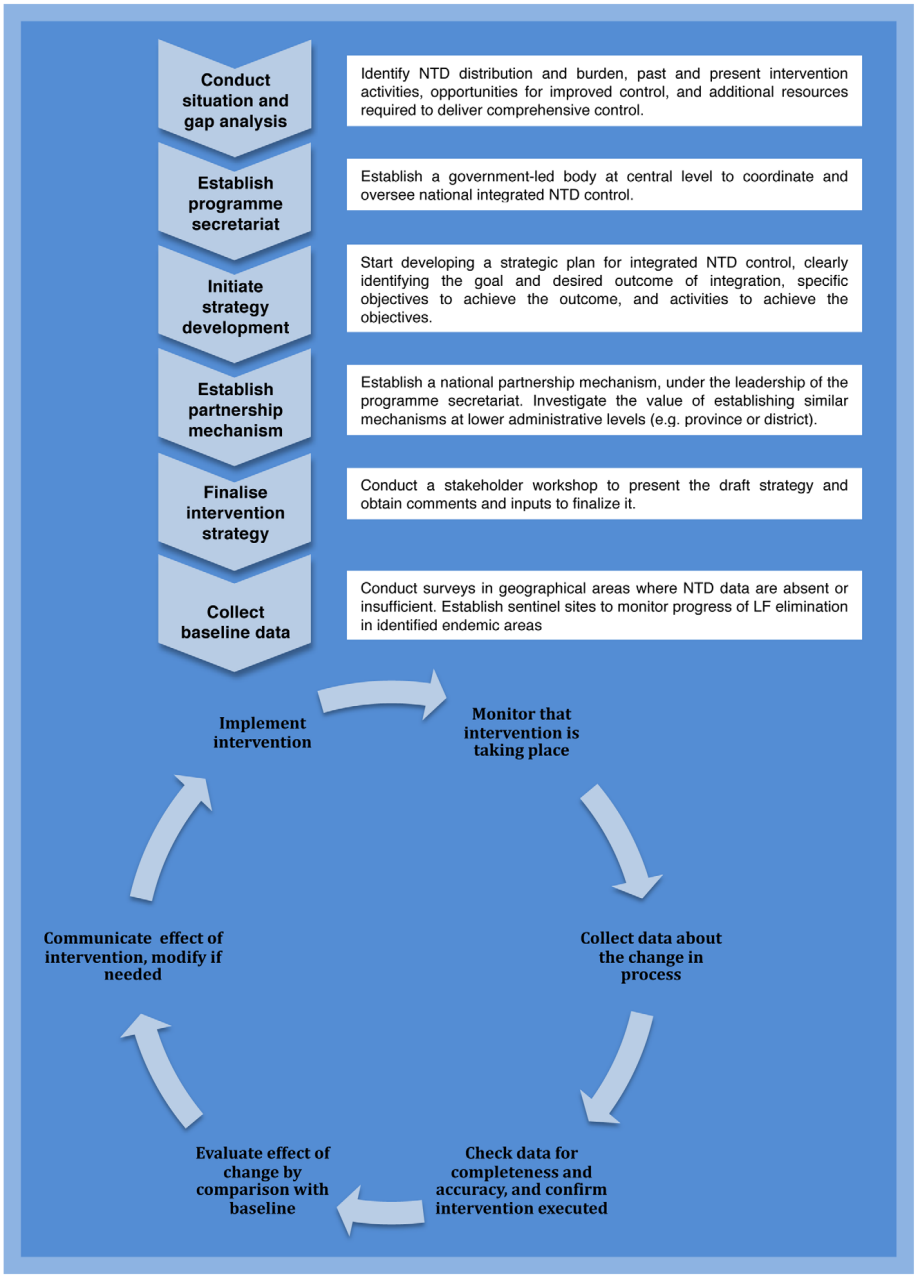
Key steps in establishing and running an integrated NTD control programme. Modified from [46].

Collection and collation of historical and current information on NTDs will provide essential background to launch the integration process (Figure 1). These data should be gathered though: (i) searches of the published and grey literature and analysis of relevant health statistics, and (ii) a situation analysis meeting by local experts. Available data can then be used to develop maps showing the distribution of the targeted diseases, highlighting areas of NTD overlap where PCT delivery may be combined and areas that remain to be surveyed. Collating this information in a detailed situation analysis (e.g. [17], [18]) will allow identification of gaps and potential means to close them, as well as provide a useful document to advocate for implementation funds.

### Where should integration be started?

The country's Ministry of Health (MoH) needs to initiate integration by deciding which departments and programmes should be part of the process. A programme secretariat or equivalent body may be formed to coordinate these, consisting of the managers of the disease-specific programmes and, where possible, representatives of the Ministry of Education and the water/sanitation sector. In our experience, the key role of the secretariat is to oversee programme implementation, with the aim of maintaining (or creating) a close link between NTD control and the health, education, and water/sanitation sector, hence preventing establishment of vertical programmes.

### What's next?

Strong partnerships between stakeholders from different sectors will be vital if the newly formed programme is to succeed in mobilizing resources, securing political commitment, and applying the strengths of the partners synergistically. A partnership mechanism, such as a NTD working group or task force, will therefore be needed to enlist and coordinate the required technical and implementation support from all stakeholders (Figure 1). To establish the partnership, the programme secretariat should approach in-country representatives of international agencies, including the World Health Organization (WHO), United Nations Children's Fund, World Food Programme and the World Bank, relevant national and international non-governmental organizations (NGOs), and key donors. In some countries it may also be possible to enlist the Water, Sanitation, and Hygiene (WASH) partnership (see http://www.wsscc.org) and/or pharmaceutical companies.

Clear terms of reference, broad participation, and flexibility to accommodate expansion will help the partnership to gain momentum. In some countries, a national stakeholder workshop has been held to facilitate the integration process. The value of such an event is greatly dependent on timing; we have found it most useful to convene a workshop to review a draft strategic plan, rather than to initiate the strategic planning process (Figure 1).

**Figure 2:**
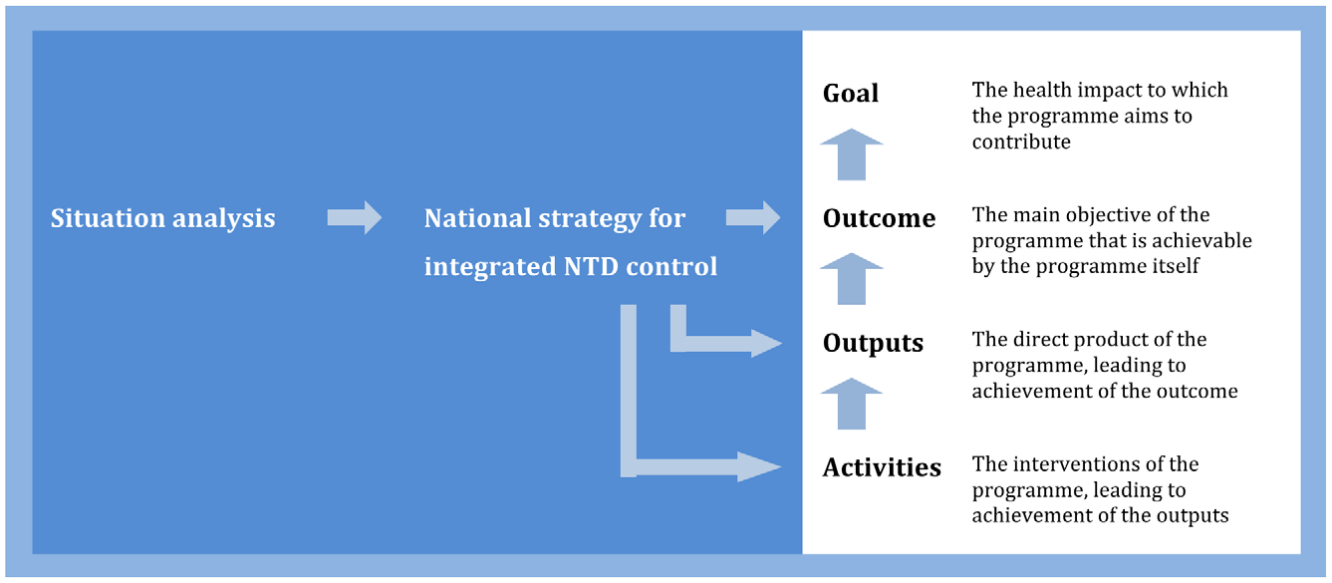
Development of a programme framework. Modified from [47].

Developing a draft strategy for discussion among NTD control partners is the first technical task for the programme secretariat. Strategic planning will require identification of the integration goal, the desired output(s) to be achieved within a specific timeframe (usually 3–5 years), and the associated objectives and activities (Figure 2). A set of generic questions may be useful for this purpose (Box 1). Though formulation of the strategy should be led by the programme secretariat, other partners need to be encouraged to contribute, particularly their experience on what is feasible with existing resources and knowledge.

**Box 1. Questions to Guide Strategy Development**

**Question 1:** Are data on the presence and prevalence of the targeted NTDs available for the whole country?
  If yes go to question 2; if not, then one of the objectives should be:
**Objective:** To develop a comprehensive map of the geographical distribution of NTDs targeted through an integrated control approach by (*specify month and year*)
**Question 2:** What delivery systems are in place? (e.g., campaigns, networks of community health workers, schools, or health facilities)
  - What is their geographic coverage?
  - What activities are they conducting and how often?
  - What are their strengths and weaknesses? (i.e., what support would be needed to deliver additional activities to avoid undermining the existing efficiency)
**Question 3:** Which of these existing delivery systems would be best suited to:
  - Sensitize communities and mobilize them to participate in control activities?
  - Conduct health education?
  - Train community health workers and other cadres of health workers?
  - Supply drugs to the periphery and pre-position them?
  - Distribute drugs to all eligible individuals on a regular basis?
  - Deliver other components of a comprehensive control strategy, such as surgery?
  - Monitor and evaluate: (i) treatment coverage, (ii) adverse drug events, (iii) impact on prevalence, intensity and morbidity, and (iv) costs?
  Depending on the answers to these questions one or more objectives to scale up interventions should be formulated. For example:
**Objective:** To increase coverage (geographical and/or population) with (*specify intervention, e.g. PCT, surgery, access to clean water*) to (*specify target, e.g. 80%*) by (*specify month and year*)

### What should be integrated and what not?

At the outset, it may seem tempting to merge all seemingly similar components of the existing NTD programmes. Health education or training materials, for example, could easily be combined into versions covering all of the targeted diseases. In our experience, however, simple health education messages rather than complicated instructions tend to work best, and training has been most effective when disease-specific materials were used. We have found that “integrated booklets” and “integrated wall charts,” providing information on transmission or control of more than one NTD, tend to be too complex for health educators and the target audience.

Few NTD programmes solely focus on integrated PCT delivery, largely because: (i) data to target interventions are often incomplete [17]–[21], making it necessary to undertake surveys, and (ii) complementary approaches, such as morbidity control, need to be scaled up simultaneously. In Nigeria and Southern Sudan, where more than one of the target diseases needed to be mapped, combined NTD surveys have been implemented [22], [23]. While this approach is efficient at classifying implementation units for LF, STH, and schistosomiasis interventions, it cannot be readily combined with the more complex population-based prevalence surveys for trachoma. However, integration of trachoma rapid assessments into above surveys could be envisaged [24]. Recent work from Nigeria also indicates that integration of trachoma into school-based surveys for schistosomiasis can identify trachoma hotspots in hypoendemic areas where school attendance is high, thus complementing population-based trachoma prevalence surveys [25].

For PCT delivery, ivermectin can be safely administered at the same time as albendazole, while praziquantel can be added only after at least one separate treatment round. Combining these drugs with azithromycin for trachoma control is currently not recommended [5]. In practice this means that increased efficiency over stand-alone PCT delivery can be immediately realized in areas endemic for onchocerciasis, LF, and STH, or for STH and schistosomiasis, and in communities that have previously received ivermectin or praziquantel. Otherwise, separate treatment rounds need to be organized and budgeted for.

The need for data and associated opportunities to integrate surveys, the possibilities to readily target communities with multiple PCT regiments, and the gaps in complementary interventions needed to ensure comprehensive disease control, all need to be scrutinized during strategy development. The decision on what and where to integrate should then be based on evidence not intuition, with new materials and guidelines being piloted before large-scale application.

### How should integrated MDA be delivered?

Whilst WHO provides technical guidance on PCT co-administration [5], operational experience is limited. The experience that exists demonstrates that a range of delivery channels can been used singly or in combination [22], [26]–[29], and need not be limited to PCT [30], [31]. There is therefore no “favourite” delivery channel, nor should programme managers look for synergies only within the NTDs. Other control programmes, for example for malaria, tuberculosis, or HIV/AIDS, may in fact be better resourced and have a wider geographical coverage than existing NTD programmes, potentially providing a stronger platform for co-implementation [32]. In practice this may mean that delivery of albendazole plus ivermectin for LF elimination is best combined with other interventions that target large geographical areas, such as distribution of long-lasting insecticidal nets [33]. Although pockets within the same area may be endemic for schistosomiasis, it may be more efficient to integrate praziquantel delivery into school-health programmes, to better target this more expensive drug and minimize its associated side effects [34].

Finally, to inform what is practical and feasible it is important to consult the target communities, as they have valuable insight into health care delivery in their own settings and how it could be improved or undermined. The success of existing NTD programmes, such as community-directed treatment with ivermectin (CDTI) for onchocerciasis control, heavily depends on community structures, customs, beliefs, and values that make programme volunteers proud and motivated [35], [36]. Modified or new delivery channels will need to recognize and build upon these factors, establishing and maintaining dialogue with communities [37].

### What should be monitored and evaluated, and how?

Integration aims to increase efficiency over stand-alone programmes. However, there is little empirical evidence to show whether and how such efficiency is achieved. Monitoring of process and evaluation of outcomes are therefore important (Box 2). From the international viewpoint, the evidence base for integrated NTD control needs to be strengthened to generate further financial support. From the country perspective, information is required to assess the success of the strategic plan and modify activities when these fail to achieve specified outputs (Figure 2). Acquiring the necessary data is challenging, partly because the target diseases each have their own goals, indicators, and methods [8], but also because existing funding is largely targeted at intervention delivery, rather than in-depth monitoring and evaluation.

**Box 2. Definition of Monitoring and Evaluation**

**Monitoring** is an ongoing process. The purpose of monitoring is to assess whether programme activities are on track and whether changes are taking place. Monitoring can be continuous or periodic. The most important aspect of monitoring is to analyse data soon after they have been collected and to use the findings to modify activities as necessary.

**Evaluation** is an overview of a programme up to a certain point in time. The purpose of evaluation is to assess whether the activities are achieving or have achieved the desired outputs and whether these outputs are likely to achieve or have achieved the desired outcome (Figure 2). Evaluations are sometimes carried out during programme implementation and should certainly be conducted at the end. Mid-term evaluation can help to identify problems that may prevent the programme achieving the desired outcome, so that appropriate changes can be made to activities and outputs. End-of-programme evaluations can assess overall success and summarize the lessons learned.

At present, most integrated NTD control programmes largely focus on measuring treatment coverage. However, if programme success is judged only on the number of people treated and the number of different drugs given, then over-treatment would make it look highly “successful” although it would actually not be cost-effective in terms of improving health. We therefore encourage development and use of additional indicators, such as parasite prevalence, intensity of infection, and morbidity indicators, so as to measure programme performance. Ideally this and other indicators should be incorporated into an integrated monitoring and evaluation platform, which could also include collection of cost data. Compilation of these additional data will ultimately allow programmes to estimate cost-effectiveness, a prerequisite for comparison of integrated NTD control with stand-alone approaches.

For LF it is already an essential programme requirement to establish sentinel sites to monitor microfilarial prevalence and other morbidity and entomological indicators during the elimination process [38]. Such sites are likely to be too few and far between to generate sufficiently detailed data to monitor the other NTDs covered here, but data collected at these sites could potentially contribute to a picture of overall disease trends.

**Figure 3:**
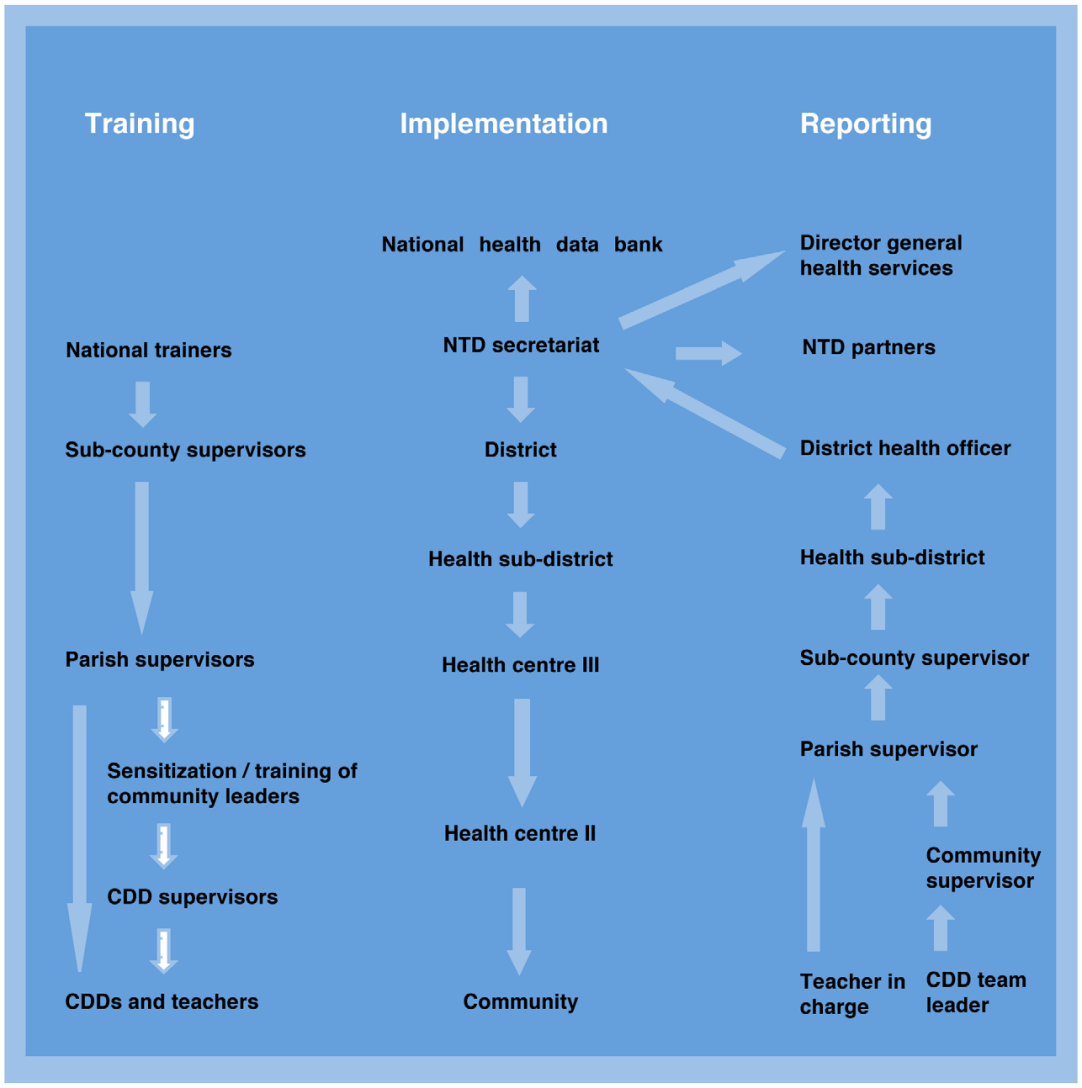
Training, implementation and reporting flow for NTDs integrated into Uganda's health system, including an alternative approach often used by NGOs (see white arrows). The training cascade is initiated by existing MoH staff at a central level, referred to as the national technical team. This team trains district trainers from subcounty or health subdistricts. These trainers then return to their duty stations to organize and coordinate training in their geographical areas. Within each area, parish supervisors and peripheral health centre staff are trained first, after which these proceed to train teachers and community volunteers. Data retrieval and reporting follows similar channels. Reports collated at the community level are delivered by parish supervisors to health centres. These data are then collected and collated by subcounty/health subdistrict coordinators into a report for the health subdistrict and submitted to the district health officer. District health officers use these reports to write a summary district report that is submitted to the NTD Secretariat in Kampala. The secretariat reports to the Director General of Health Services, distributes copies of the report to partners, and submits the data to the national data bank. The same system and staff are use to report on other community-based activities, such as TB, leprosy, malaria, and HIV programmes.

WHO provides disease-specific reporting formats and is developing guidelines on monitoring and evaluation for integrated NTD control programmes. Specific guidance on how to evaluate the epidemiological impact of national helminth control programmes has been provided elsewhere [39] and additional tools have been developed by the NTD Initiative (www.neglecteddiseases.gov/resources/tools_guidelines/index.html) led by the U.S. Agency for International Development. The NTD Secretariat could use these resources to develop simple standardised reporting forms to record coverage data and to design surveys that specifically measure outcome and impact. To avoid establishment of a separate system, reporting forms and procedures should be consistent with routine MoH operations. The example of Uganda's routine system is provided in Figure 3.

### What are the challenges?

Effective NTD control requires multi-pronged strategies including treatment, health education, provision of clean water and sanitation, and surgery. Activities other than PCT are often more costly and difficult to scale up, making donors less inclined to invest in them [40], [41]. Countries wishing to embark on integrated NTD control thus face the challenge of implementing a comprehensive control strategy in an environment where most external resources are focused on only one component—integrated PCT delivery. This focus is the result of substantial global advocacy on the potential benefits of PCT integration, which has been extremely successful at raising the NTD profile in general. As a result, however, integration of PCT delivery seems to have been transformed from an activity into a key objective. Programme managers may thus be tempted or pressured to pursue this “objective,” rather than to balance integrated and disease-specific approaches in the interest of efficiency [15].

At the implementation level, integration inevitably puts an additional strain on existing systems. This may have no effect on quality where there is spare capacity. For systems already struggling to meet their targets, however, integration can be detrimental. For example, community volunteers may feel overburdened by the delivery of additional drugs, leading to considerable attrition if demands for payment or additional staff are not met [42]–[44]. A major implementation challenge is therefore to find the optimal balance between available inputs and well-defined and achievable outputs of delivery systems for NTD control.

### How can these challenges be avoided or overcome?

Successful establishment and running of an integrated NTD control programme hinges on effective partnership and implementation of a stepwise and evidence-based approach, allowing many of the immediate challenges to be minimized or avoided. More specifically, forging close in-country partnerships with relevant ministries and international agencies will increase technical and material programme contributions. Such coordination and collaboration between sectors and partners may, for example, allow targeting of activities on education, water development, and eye care to areas where PCT delivery is ongoing or planned, hence allowing implementation of the full SAFE strategy for trachoma control [45]. Pooling of resources for service delivery should also increase the programme's ability to support delivery structures in terms of community consultation, recruitment, and adequate training of additional volunteers to decrease the workload of the individual, thus minimizing attrition.

Clearly there are key lessons to be learned from the last 5 years of scaling up integrated NTD control (Box 3). With the anticipated vast increase in financial support for NTD control from the US government in 2011 (http://www.state.gov/documents/organization/135888.pdf), it will be crucial that these lessons be applied to future programming; amongst other improvements this would ensure that “integration” reverts to the status of an activity aimed at maximising efficiency rather than being seen as an end in itself.

**Box 3. Key Learning Points**

- **Integration** of NTD control programmes **should be led by government** (e.g., an NTD Secretariat), to ensure that the approach is consistent with overall health systems development and does not lead to the establishment of stand-alone programmes.
- **Clear structures for coordination, implementation, and reporting** from centre to periphery should be developed by the NTD Secretariat with input from implementing partners, clarifying who is responsible for what. Formation of NTD coordination mechanisms (e.g., task force) at central and district levels is essential to support implementation.
- A **comprehensive national strategy** should be developed at the outset, clearly outlining the goal, outcome, objectives, and activities of the integrated NTD control programme over a specified timeframe of 3–5 years. The strategy should provide information on structures to be used for coordination and implementation, and the indicators used to monitor progress and outcomes.
- WHO recommends comprehensive strategies for the control of each NTD targeted by an integrated approach. Although each of these strategies includes PCT, **other approaches to prevention and case-management are equally important** and should be supported.
- **Coverage data should not be the only indicator** to monitor integrated NTD control programmes and to judge their success, particularly as these data provide an incentive to distribute large quantities of drugs regardless of actual need.
